# Electroconvulsive therapy for trunk and visceral cenesthopathy associated with major depressive disorder: A case series of three elderly women

**DOI:** 10.1002/pcn5.70438

**Published:** 2026-10-30

**Authors:** Yoshikazu Masuda, Ken Wada, Kaoru Chikurinji, Hirotoshi Ri

**Affiliations:** ^1^ Department of Psychiatry and Neuroscience Hiroshima University Hospital Hiroshima Japan; ^2^ Department of Psychiatry Hiroshima Citizens Hospital, Hiroshima City Hospital Organization Hiroshima Japan; ^3^ Department of Psychiatry and Neuroscience Hiroshima Prefectural Hospital Hiroshima Japan; ^4^ Department of Neuropsychiatry Okayama University Hospital Okayama Japan

**Keywords:** cenesthopathy, electroconvulsive therapy, major depressive disorder, visceral cenesthopathy

## Abstract

**Background:**

Severe cenesthopathy associated with major depressive disorder (MDD) may cause malnutrition and functional decline. We report three elderly women with trunk and visceral cenesthopathy associated with MDD who improved markedly following electroconvulsive therapy (ECT).

**Case Presentation:**

**Case A:** A woman in her 60s developed MDD while caring for her mother with dementia. Despite pharmacological treatment, trunk, limb, and oral cenesthopathy developed 6 months later, resulting in reduced food intake and physical deterioration. Symptoms improved after six ECT sessions, nearly resolved after 12, and ECT was completed after 15 sessions without recurrence.

**Case B:** A woman in her 70s developed MDD after prolonged caregiving stress. Despite pharmacological treatment, she developed trunk and visceral cenesthopathy with organ displacement sensations, dyspnea, speech disturbance, and loss of appetite. Cenesthopathy resolved after three ECT sessions; dyspnea and speech disturbance improved after 10. ECT was completed after 13 sessions. After recurrence, a second acute ECT course followed by maintenance ECT maintained clinical stability.

**Case C:** A woman in her 70s with longstanding MDD developed visceral and oral cenesthopathy after discontinuing antidepressants. Despite pharmacological treatment, she became unable to eat or drink. Cenesthopathy resolved after four ECT sessions, restoring oral intake. ECT was completed after eight sessions without recurrence.

**Conclusion:**

ECT may be an effective treatment for trunk and visceral cenesthopathy associated with MDD. Early consideration of ECT may be warranted when malnutrition or physical deterioration necessitates rapid symptom control.

## BACKGROUND

Cenesthopathy is a pathological condition characterized by persistent and bizarre complaints of abnormal bodily sensations in the absence of objective physical findings that adequately explain the symptoms. First described by Dupré and Camus in 1907, it remains a classic psychopathological concept.^1^ Although it is not recognized as an independent diagnostic entity in contemporary classification systems such as the DSM‐5 or ICD‐11, the term continues to be widely used in Japan, particularly in psychiatry and oral and maxillofacial surgery. The term encompasses not only monosymptomatic cases but also pathological bodily sensations associated with psychiatric disorders such as schizophrenia, major depressive disorder (MDD), and organic mental disorders.

Clinically, cenesthopathy is often categorized according to the predominant site of symptoms into oral cenesthopathy and trunk and visceral cenesthopathy. The condition frequently follows a chronic course, and severe cases may result in malnutrition, functional decline, and physical deterioration. Although several case reports have described successful treatment with electroconvulsive therapy (ECT), most have involved oral cenesthopathy, and reports of ECT for trunk and visceral cenesthopathy remain scarce.

Here, we report three elderly women with trunk and visceral cenesthopathy associated with MDD who showed marked clinical improvement following ECT.

## CASE PRESENTATION

### Patient A

Patient A was a woman in her 60s who developed MDD while caring for her mother with dementia. Despite treatment with duloxetine 60 mg/day, her depressive symptoms persisted. Around the same time, she developed low back pain, but orthopedic evaluation revealed no organic abnormalities. Approximately 6 months after the onset of low back pain, she began to complain of abnormal sensations in the lower back and upper limbs describing them in bizarre terms, such as “There are two lumps in my lower back, and they send electricity through my body, making my hands numb,” as if some animate entity were present within her body. She also complained of hypersalivation, resulting in markedly reduced food intake.

She spent most of the day lying prone in bed and required assistance with meals. She was admitted to our department because of severe functional decline. Cenesthopathy involving the limbs, trunk, and oral cavity and depressive symptoms persisted despite duloxetine 60 mg/day combined with brexpiprazole 2 mg/day. Progressive physical deterioration prompted ECT. After approximately six sessions, her cenesthopathy had diminished to a simple complaint of low back pain, and she regained mobility. After twelve sessions, her symptoms had almost completely disappeared, and she resumed normal daily activities. ECT was completed after fifteen sessions. No serious adverse events were observed. After discharge, duloxetine 60 mg/day and brexpiprazole 2 mg/day were continued. During 5 years of follow‐up, she remained clinically stable without recurrence of either cenesthopathy or depressive symptoms.

### Patient B

Patient B was a woman in her 70s with a history of mild depression who developed recurrent MDD after prolonged verbal and physical abuse by her mother with dementia. Despite treatment with sertraline 100 mg/day, she subsequently developed bizarre bodily sensations, including visceral displacement and abnormal dyspnea, with statements such as “My stomach is moving inside my body” and “Air is leaking from my body, so I cannot breathe.” She also developed nonfluent speech and reduced food intake.

Gastrointestinal investigations revealed no abnormalities, and brain MRI showed only mild chronic ischemic changes. Pharmacotherapy with mirtazapine 30 mg/day and aripiprazole 12 mg/day failed to achieve sufficient clinical improvement. Severe agitation and functional impairment led to her admission to our department.

After initiation of ECT, the sensation of visceral displacement resolved after three sessions, and insomnia improved. Dyspnea, speech disturbance, anxiety, and agitation markedly improved by the 10th session. ECT was completed after 13 sessions without adverse events. Six months after discharge, depressive symptoms and cenesthopathy recurred. She underwent a second acute course of right unilateral (RUL) ECT, followed by monthly maintenance RUL ECT. Mirtazapine 30 mg/day and aripiprazole 12 mg/day were continued. At the latest follow‐up, 5 years after the initiation of monthly maintenance RUL ECT, treatment was ongoing. Mild cenesthopathic symptoms persisted without impairment of physical functioning or activities of daily living, and depressive symptoms had not recurred.

### Patient C

Patient C was a woman in her 70s with longstanding MDD associated with work‐related and caregiving stress. After discontinuing medication on her own, she developed epigastric discomfort. Gastrointestinal examinations revealed no organic abnormalities. Approximately 6 months later, she began complaining of bizarre visceral and oral sensations, including “My intestines are becoming hard,” “My tongue moves on its own,” and “My internal organs are contracting.” She became unable to eat or drink and developed severe physical deterioration.

Treatment with escitalopram 20 mg/day and aripiprazole 12 mg/day failed to achieve sufficient clinical improvement, and progressive malnutrition necessitated intravenous fluids and nasogastric tube feeding. She was therefore referred to our department for ECT. Visceral and oral cenesthopathy completely resolved after four ECT sessions, accompanied by recovery of oral intake, motivation, and daily functioning. ECT was completed after eight sessions without adverse events. Escitalopram and aripiprazole were gradually tapered and discontinued 2 years after ECT. Neither cenesthopathy nor depressive symptoms recurred, and psychiatric follow‐up ended 3 years after ECT.

## ECT PROCEDURE

In all three patients, ECT was administered three times weekly using a Thymatron System IV (pulse width, 0.5 ms) under general anesthesia with succinylcholine. Propofol (50 mg) was used for Patient A and thiopental (75 mg) for Patients B and C. Patient A received bitemporal ECT for the first two sessions at an initial stimulus dose of 35% (176 mC; half‐age method). Because of an increased seizure threshold, treatment was switched to RUL ECT from the third session. The RUL seizure threshold was 5%, and treatment began at 30% (151 mC; six times the seizure threshold) and subsequently increased to 100%. A total of 15 ECT sessions were administered. Patient B received 13 bitemporal ECT sessions during the first acute course, with the stimulus dose increased from 35% (176 mC; half‐age method) to 70%. Following relapse, she received 16 RUL ECT sessions; the seizure threshold was 5%, and treatment began at 15% (76 mC; three times the seizure threshold) and increased to 30%. This was followed by monthly maintenance RUL ECT at 30%. Patient C received eight bitemporal ECT sessions at 35% (176 mC; half‐age method) without dose escalation. No pharmacological seizure augmentation techniques, such as caffeine or theophylline, were used in any of the three patients.

## DISCUSSION

In all three patients, pharmacotherapy with antidepressants combined with antipsychotics failed to achieve sufficient improvement in cenesthopathy, and no clear clinical improvement in other depressive symptoms was observed. Persistent symptoms were accompanied by marked functional decline, reduced oral intake, and progressive physical deterioration. Following the initiation of ECT, both cenesthopathy and depressive symptoms improved rapidly. The ECT parameters differed among the three patients. In Patient A, electrode placement was switched from bitemporal to RUL ECT because of an increased seizure threshold. Previous study has suggested that switching from bilateral to RUL electrode placement may facilitate seizure induction in patients with high seizure thresholds.^2^ Patient B responded to both bitemporal and RUL ECT, followed by monthly maintenance RUL ECT, whereas Patient C responded to bitemporal ECT without dose escalation. No serious adverse events were observed. However, because of the small sample and absence of systematic cognitive assessments, associations between specific ECT parameters and efficacy or cognitive adverse effects cannot be established.

Cenesthopathy is a classic psychopathological concept first described by Dupré and Camus in 1907.^1^ Because it is not recognized as an independent diagnostic entity in contemporary classification systems such as the DSM‐5 or ICD‐11, similar phenomena are often described as *somatic delusions*, *hypochondriacal delusions*, or *somatic hallucinations*. However, the boundaries among these concepts remain unclear, and considerable phenomenological overlap exists. In the present report, we use the term *cenesthopathy* because the patients' principal complaints consisted of bizarre and difficult‐to‐explain bodily sensations rather than fixed delusional interpretations.

Several case reports have suggested that ECT is effective for cenesthopathy, including oral cenesthopathy without psychiatric illness,^3^ oral cenesthopathy associated with MDD,^4^, ^5^ esophageal or abdominal cenesthopathy associated with depression,^6^, ^7^ oral cenesthopathy associated with bipolar disorder,^8^ and abdominal, lower limb, or cranial cenesthopathy associated with schizoaffective disorder.^9^, ^10^, ^11^ However, most published reports have focused on oral cenesthopathy, and evidence regarding trunk and visceral cenesthopathy remains limited. Furthermore, only a small number of patients have been described, and the therapeutic efficacy of ECT for trunk and visceral cenesthopathy has not been systematically evaluated. Previous neuroimaging studies have suggested that ECT may improve regional cerebral blood flow abnormalities observed in patients with cenesthopathy, although the precise mechanisms remain unclear.^4^, ^11^

The three patients presented here shared several notable clinical characteristics. All were elderly women with MDD who developed cenesthopathy predominantly involving the trunk, limbs, or viscera despite pharmacological treatment. All had experienced substantial psychosocial stress, particularly caregiver burden, before symptom exacerbation. Cenesthopathy was accompanied by rapid functional decline, reduced oral intake, and physical deterioration requiring rapid symptom control. Although trunk and visceral symptoms predominated, Patients A and C also exhibited oral cenesthopathy, suggesting that these symptom distributions may coexist rather than represent distinct clinical entities.

Previous reviews have reported that cenesthopathy associated with schizophrenia is more common in younger patients, whereas depression‐ and organic brain disease‐associated cenesthopathy predominates in older adults.^12^ A review of 606 patients with oral cenesthopathy further demonstrated that MDD was the most common psychiatric comorbidity, accounting for 24.69% of cases, and suggested that dental treatment during the acute phase of depression may increase the risk of oral cenesthopathy.^13^ Another study reported that trunk and visceral cenesthopathy is more common in younger men, whereas oral cenesthopathy predominantly affects elderly women^12^ (Figure 1). In contrast, our patients were all elderly women with predominantly trunk and visceral symptoms, indicating that this clinical presentation may be more heterogeneous than previously recognized.

**Figure 1:**
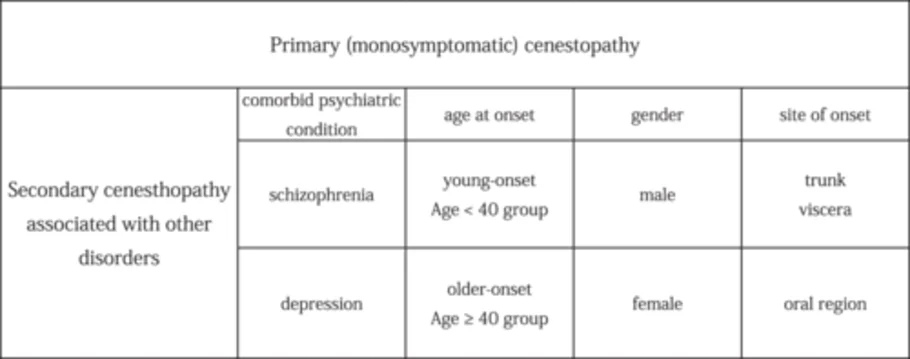
Classification of cenesthopathy. Cenesthopathy is broadly classified into primary (monosymptomatic) and secondary forms. Secondary cenesthopathy occurs in association with underlying psychiatric disorders, and the predominant distribution of symptoms varies according to the psychiatric disorder, age, and sex.

Various pharmacological treatments for cenesthopathy have been reported, including antidepressants such as paroxetine, fluvoxamine, and amitriptyline, and antipsychotics such as sulpiride, risperidone, olanzapine, aripiprazole, and perospirone. However, treatment response is highly variable, and no standardized therapeutic strategy has been established.^13^ In our three patients, pharmacological treatment failed to achieve sufficient clinical improvement before progressive malnutrition and physical deterioration developed, making rapid symptom control with ECT clinically necessary.

Non‐pharmacological approaches, including psychoeducation and cognitive behavioral therapy (CBT), have also been employed. In particular, CBT aims not necessarily to eliminate abnormal bodily sensations but rather to reduce excessive preoccupation and catastrophic interpretations while improving daily functioning despite persistent symptoms.^14^ However, in patients such as ours, who develop severe malnutrition and rapid physical deterioration because of cenesthopathy, rapid symptom resolution is essential. In such situations, prolonged trials of pharmacotherapy or psychotherapy alone may not be sufficient.

Our findings suggest that ECT should be considered early for patients with severe cenesthopathy associated with MDD when progressive malnutrition or physical deterioration necessitates rapid symptom control. Further studies involving larger cohorts are required to clarify the clinical characteristics of patients most likely to benefit from ECT and to elucidate the neurobiological mechanisms underlying its therapeutic effects.

## CONCLUSION

ECT may be an effective treatment option for patients with trunk and visceral cenesthopathy associated with MDD. Particularly in older adults, severe cenesthopathy may lead to malnutrition, functional decline, and progressive physical deterioration. Early consideration of ECT may therefore be warranted when rapid symptom control is required.

## AUTHOR CONTRIBUTIONS

Yoshikazu Masuda drafted and revised the manuscript. All other authors contributed to patient management and manuscript revision. All authors approved the final version of the manuscript.

## CONFLICT OF INTEREST STATEMENT

The authors declare no conflicts of interest.

## ETHICS APPROVAL STATEMENT

Ethical approval was not required for this case report in accordance with local guidelines. Written informed consent was obtained from all patients.

## PATIENT CONSENT STATEMENT

The authors obtained written informed consent from the patient for publication of this case report.

## CLINICAL TRIAL REGISTRATION

N/A.

## Data Availability

The data that support the findings of this study are available on request from the corresponding author. The data are not publicly available due to privacy or ethical restrictions.
